# Organocatalyst treatment improves variant calling and mutant detection in archival clinical samples

**DOI:** 10.1038/s41598-022-10301-0

**Published:** 2022-04-20

**Authors:** Leah C. Wehmas, Charles E. Wood, Ping Guan, Mark Gosink, Susan D. Hester

**Affiliations:** 1 Office of Research and Development, U.S. Environmental Protection Agency, MD-B105-03, 109 T.W. Alexander Drive, Research Triangle Park, NC USA; 2 National Cancer Institute, Bethesda, MD USA; 3 Pfizer, Groton, CT USA; 4 Present Address: Boehringer Ingelheim Pharmaceuticals, Inc., Ridgefield, CT USA

**Keywords:** Chemical modification, Chemical genetics, Cancer genomics

## Abstract

Formalin fixation of biological specimens damages nucleic acids and limits their use in genomic analyses. Previously, we showed that RNA isolation with an organocatalyst (2-amino-5-methylphenyl phosphonic acid, used to speed up reversal of formalin-induced adducts) and extended heated incubation (ORGΔ) improved RNA-sequencing data from formalin-fixed paraffin-embedded (FFPE) tissue samples. The primary goal of this study was to evaluate whether ORGΔ treatment improves DNA-sequencing data from clinical FFPE samples. We isolated RNA and DNA ± ORGΔ from paired FFPE and frozen human renal and ovarian carcinoma specimens collected as part of the National Cancer Institute Biospecimen Pre-analytical Variables program. Tumor types were microscopically confirmed from adjacent tissue sections. Following extraction, DNA was fragmented and sequenced and differences were compared between frozen and FFPE sample pairs. Treatment with ORGΔ improved concurrent SNP calls in FFPE DNA compared to non-ORGΔ FFPE samples and enhanced confidence in SNP calls for all FFPE DNA samples, beyond that of matched frozen samples. In general, the concordant SNPs identified in paired frozen and FFPE DNA samples agreed for both genotype and homozygosity *vs*. heterozygosity of calls regardless of ORGΔ treatment. The increased confidence in ORGΔ FFPE DNA variant calls relative to the matched frozen DNA suggests a novel application of this method. With further optimization, this method may improve quality of DNA-sequencing data in FFPE as well as frozen tissue samples.

## Introduction

Global biorepositories house hundreds of millions of archived tissue specimens. These unique collections are curated by a diverse range of organizations, from hospitals and academic institutions to museums and government research laboratories, and often represent the only remaining biological archive from rare case studies or expensive experimental studies and clinical trials. The vast majority of archived tissue specimens are initially preserved in formalin and then stored as formalin-fixed paraffin-embedded (FFPE) blocks. The process of formalin fixation includes extensive cross-linking and other biochemical modifications that preserve microscopic morphology but interfere with molecular analyses. These changes reduce the amount of intact nucleic acid and result in sequencing artifacts, reducing the reliability of genomic data from FFPE samples^1–4^. To address this issue, improved tools are needed to increase nucleic acid yield, reduce artifacts, and facilitate analysis and interpretation of genomic data specifically from formalin-fixed samples. Better methods for sequencing of DNA from FFPE tissue samples would enhance retrospective mining of human tumor banks, targeted mutational and single nucleotide polymorphism (SNP) analyses, concordance studies with immunohistochemical and in situ hybridization biomarkers, patient selection for clinical trials, and related precision medicine initiatives.

Recently, we demonstrated that relatively simple changes to standard RNA isolation protocols using an organocatalyst with extended heated incubation (ORGΔ) significantly improved the quality of RNA-sequencing (RNA-seq) data from FFPE samples^5,6^. The original study describing the bi-functional organocatalyst suggested that it preferentially removed formaldehyde adducts from all nucleic acids, but its effectiveness on DNA sequencing results was not assessed^6^. In the present study, we hypothesized that use of this same ORGΔ method would also enhance FFPE DNA data quality, increasing detection and reliability of variant calls for mutation analyses of tumor samples. Furthermore, as RNA transcripts are essentially an edited, condensed version of the expressed genome, we predicted that variants in RNA-seq data would be present in both the RNA-seq data and gene coding regions of the DNA-seq data. To test these ideas, we analyzed paired frozen (FR) and FFPE samples from human kidney and ovarian cancer specimens collected through the Biospecimen Pre-analytical Variables (BPV) program, a National Cancer Institute (NCI)-sponsored study that systematically assessed the effects of pre-analytical factors on the molecular profile of biospecimens^7^.

We applied ORGΔ to FFPE RNA and DNA samples prior to total RNA-seq and DNA Exome-seq, respectively, and analyzed the results relative to FR sample controls to detect changes in variant analysis. Our results show that ORGΔ improved FFPE DNA Exome-seq data quality in several ways, including higher-quality SNP calls. These findings should have important applications in translational science and precision medicine.

## Results

### ORGΔ effects on RNA/DNA yield and quality measures

Fixation and time in formalin resulted in lower RNA yield after normalization to approximate cellularity. For example, 12-h formalin fixation (with ORGΔ) resulted in 2.0- and 1.2-fold less RNA for kidney and ovary, respectively, compared to their matched OCT FR controls (1.5 × 10^–5^ ± 1.9 × 10^–6^ (SE) and 2.3 × 10^–5^ ± 1.0 × 10^–5^ µg/cell, respectively), while 72-h formalin fixation (with ORGΔ) resulted in 2.0-fold less RNA compared to OCT FR ovary (Supplementary Table 1). This reduction in RNA yield with increased time in formalin observed in the ovary samples is consistent with results from other studies.

Yields tended to be higher for RNA compared to DNA for matched ORGΔ FFPE tissue samples (kidney and ovary). On average, we obtained 6.3-fold less DNA from 12-h ORGΔ FFPE kidney samples (1.2 × 10^–6^ µg/cell) and 7.3- and 9.6-fold less DNA from 12-h and 72-h ORGΔ FFPE ovary samples (2.6 × 10^–6^ and 1.2 × 10^–6^ µg/cell) compared to RNA, respectively (Supplementary Fig. 1, Supplementary Table 2). (Note that these comparisons did not account for the change in ORGΔ incubation temperature for FFPE DNA.) Furthermore, isolation of DNA from FFPE tissue samples tended to require a greater number of tissue sections (~ 6–10 × 10 μm) as input compared to the number of tissue sections required for isolation of RNA (~ 4–6 × 10 μm). For ovary, the longer fixation time (ORGΔ 72-h FFPE) resulted in 2.2-fold lower DNA yield compared to ORGΔ 12-h FFPE samples; however, this difference in yield may have been influenced by differences in incubation temperatures applied during DNA isolation. While we could not systematically investigate the influence of ORGΔ incubation temperature on DNA isolation amount because of sample limitations, we did observe that increasing the incubation temperature from 55 to 70 °C can improve DNA recovery for exome sequencing library preparation, especially for ORGΔ 72-h FFPE ovary, in which the higher incubation temperature resulted in 9.8-fold higher yields (Supplementary Fig. 1). Further work is needed to identify the optimal ORGΔ incubation temperature for FFPE DNA.

The effects of ORGΔ on RNA quality in FFPE samples were mixed. For example, the average RNA integrity number (RIN) value was 6.2 for OCT FR kidney samples and 2.1 for 12-h ORGΔ FFPE samples. In contrast, the RIN values for OCT FR ovary samples were lower than OCT FR kidney samples and did not change much, on average, with time in formalin (RIN values of 2.2 and 3.2 for 12-h and 72-h ORGΔ FFPE samples, respectively) (Supplementary Table 1). DNA integrity values were not collected for the kidney and ovary samples.

Following sequencing, there were few significant differences for the typical pre-alignment RNA quality metrics. Significant group level differences in ovary (p-value = 0.04) were identified for nucleotide content, suggesting a shift with fixation that resulted in higher %A and %Poly A/T and lower %G, %C, and %GC bases across reads, but these differences did not extend to significance on pairwise comparisons. Average insert size between sequencing adapters and changes in the percent of duplicated reads also showed significant group level differences for ovary (p-value = 0.02 and 0.04, respectively) that also did not extend to pairwise comparisons. With 12-h formalin fixation, ORGΔ FFPE kidney insert size was 1.2-fold lower relative to OCT FR samples (158.1 ± 1.5 base pairs). A similar effect was seen in 12-h and 72-h ORGΔ FFPE ovary samples, which were 1.3- and 1.4-fold lower compared to OCT FR samples (175.0 ± 14.0 base pairs). The percentage of average duplicated reads tended to increase with time in formalin for RNA. This was observed 12-h ORGΔ FFPE kidney (45.8 ± 16.2%) and 72-h ORGΔ FFPE ovary (50.2 ± 11.7%) samples relative to respective, paired OCT FR controls (38.8% and 44.4%). Forward strand read 1 tended to have higher percentages of duplicated reads compared to reverse strand read 2 regardless of fixation, but the formalin-related increases in % duplicates were slightly stronger in read 2 (Supplementary Table 3).

For DNA, the OCT FR samples were sequenced by collaborators at a different time. Therefore, it was not possible to make direct comparisons across DNA pre-alignment metrics to OCT FR samples. However, pre-sequencing and pre-alignment quality information on FFPE (without ORGΔ treatment) and OCT FR DNA samples is available in Ref.^7^. The DNA pre-alignment quality metrics were relatively consistent across ORGΔ FFPE samples. Shifts in nucleotide content between 12 and 72-h ORGΔ FFPE ovary DNA was not detected. As with ORGΔ FFPE RNA, time in formalin tended to reduce the average insert size for ovary samples from 151.8 to 144.4 base pairs at 12-h and 72-h, respectively. The average insert size for DNA library preparations from 12-h ORGΔ FFPE kidney samples was similar to time in formalin matched ovary samples at 154.5 base pairs (Supplementary Table 4). When accounting for differences in ORGΔ incubation temperature, 70 °C resulted in slightly larger (1.1-fold) insert size for both 12-h and 72-h ORGΔ FFPE ovary DNA compared to 55 °C incubated samples (Supplementary Table 5). There was a potential difference between total read clusters generated for kidney and ovary DNA (ranging from 73.5 to 59.5 mil) but this coincided with the amount of DNA submitted for sequencing. The percent of duplicated reads in ORGΔ FFPE ovary DNA was higher with increased time in formalin 41.4 to 53.2% at 12-h and 72-h, respectively. Percent duplicate reads in 12-h ORGΔ FFPE kidney was similar to that of ovary at 41.0%). Interestingly, forward strand read 1 showed higher % duplicates than reverse strand read 2 similar to RNA-seq results regardless of tissue (Supplementary Table 4). The effects of ORGΔ incubation temperature on duplicated reads were mixed (Supplementary Table 5).

### Effects of FFPE and ORGΔ on SNPs

**Figure 1:**
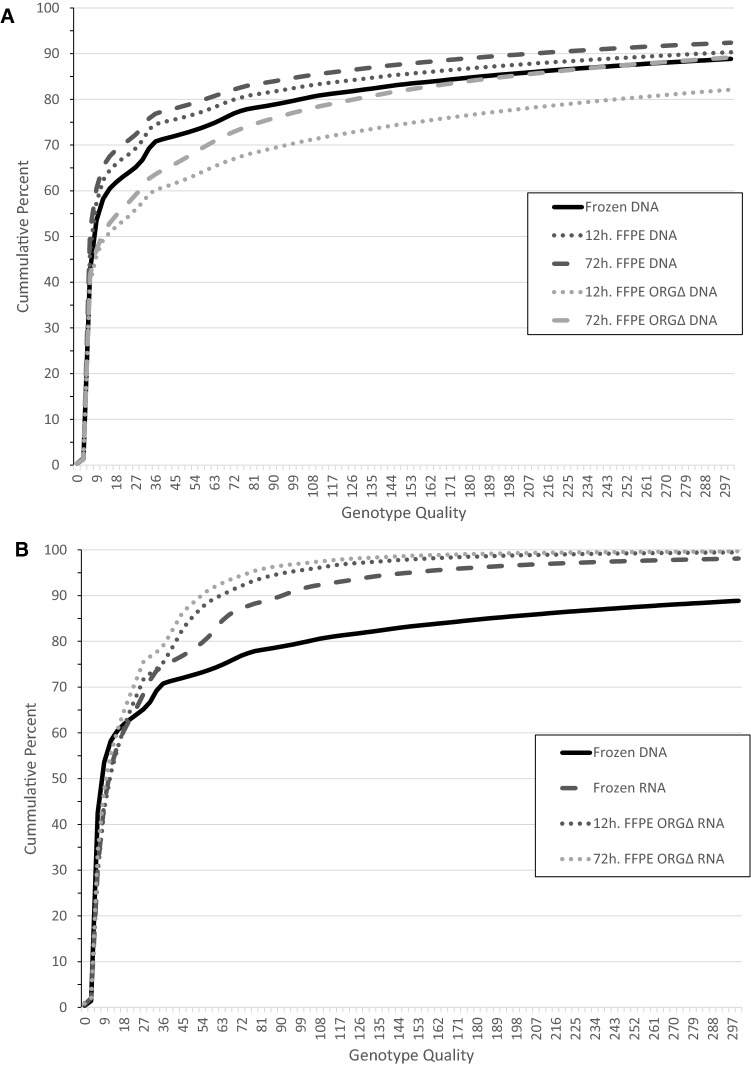
Influence of organocatalyst (ORG∆) treatment on cumulative percent SNP genotype quality (GQ) scores. GQ scores for the indicated sets of samples were extracted from the VCF files generated by GATK. The percent of SNPs for each sample type scoring at or below the indicated scores on the x-axis are displayed on the y-axis. (**A**) SNP GQ scores from DNA samples that had been frozen (FR) or formalin-fixed for 12-h or 72-h (FFPE), with or without ORG∆. (**B**) SNP GQ scores from FR DNA, FR RNA, ORG∆-treated 12-h FFPE RNA, and ORG∆-treated 72-h FFPE RNA samples. The FR and 12-h FFPE samples used sections from kidney and ovary tumors, whereas 72-h FFPE samples used sections from only the ovary tumors.

Following alignment, SNP quality was evaluated by comparing the GQ scores generated by GATK. SNPs from patient-matched DNA data were compared between different preservation conditions (12-h FFPE, 72-h FFPE, 12-h ORGΔ FFPE, and 72-h ORGΔ FFPE), relative to patient-matched OCT FR control samples (Fig. 1A). SNPs from patient matched RNA data were compared between 12-h ORGΔ FFPE and 72-h ORGΔ FFPE relative to OCT FR RNA with FR DNA included as reference (Fig. 1B). For example, for the FR DNA *vs*. 12-h FFPE DNA comparison, SNPs from the FR DNA sample of patient 84 were only compared to the SNPs from 12-h FFPE samples of patient 84. GQ scores from each comparison were summarized by each treatment group with data from matched preservations (ovary and kidney FR, 12-h FFPE and 12-h ORGΔ FFPE) initially combined. As shown in Fig. 1A and Table 1, DNA extracted from samples stored in FFPE blocks displayed a leftward shift toward a greater percentage of SNPs at poorer GQ scores compared to OCT FR samples. When separated by tissue, DNA from 12-h FFPE ovary had poorer GQ scores than 12-h FFPE kidney (Supplementary Fig. 2). Treatment of FFPE samples with ORGΔ resulted in a higher proportion of SNPs with better GQ scores, even better than OCT FR DNA samples for both tissue types. RNA displayed a different trend with ORGΔ treatment compared to DNA. RNA from ORGΔ treated FFPE samples had poorer SNP GQ scores than OCT FR rather than better that trended toward worse with longer times in formalin. Moreover, the RNA SNP GQ scores never reach that of the FR DNA samples (Fig. 1B, Table 1).

**Table 1:** p-values from Kolmogorov–Smirnov (KS) tests of genotype quality scores.

	DNA FFPE	DNA FFPE 12-h	DNA FFPE 72-h	DNA FFPE ORG∆	DNA FFPE 12-h ORG∆	DNA FFPE 72-h ORG∆
DNA FR	1.39e−03	3.79e–02	3.32e−08	1.46e−12	3.66e−15	7.10e−01

	RNA FR	RNA FFPE ORG∆	RNA FFPE 12-h ORG∆	RNA FFPE 72-h ORG∆		
DNA FR	2.22e−16	2.22e−16	2.22e−16	2.22e−16		
RNA FR		4.46e−12	1.09e−10	4.44e−16		

Two-sided KS tests were performed between the genotype quality (GQ) scores indicated pairs of samples. Each sample consisted of the continuous distribution of the percent of SNPs for each sample scoring at or below the indicated GQ score. ORG∆ indicates organocatalyst-treated samples. “FFPE” consists of combined data from 12-h kidney FFPE, 12-h ovary FFPE, and 72-h ovary FFPE samples, “12-h FFPE” consists of combined data from 12-h kidney and ovary FFPE samples, and “72-h FFPE” consists of data only from 72-h ovary FFPE samples.

**Figure 2:**
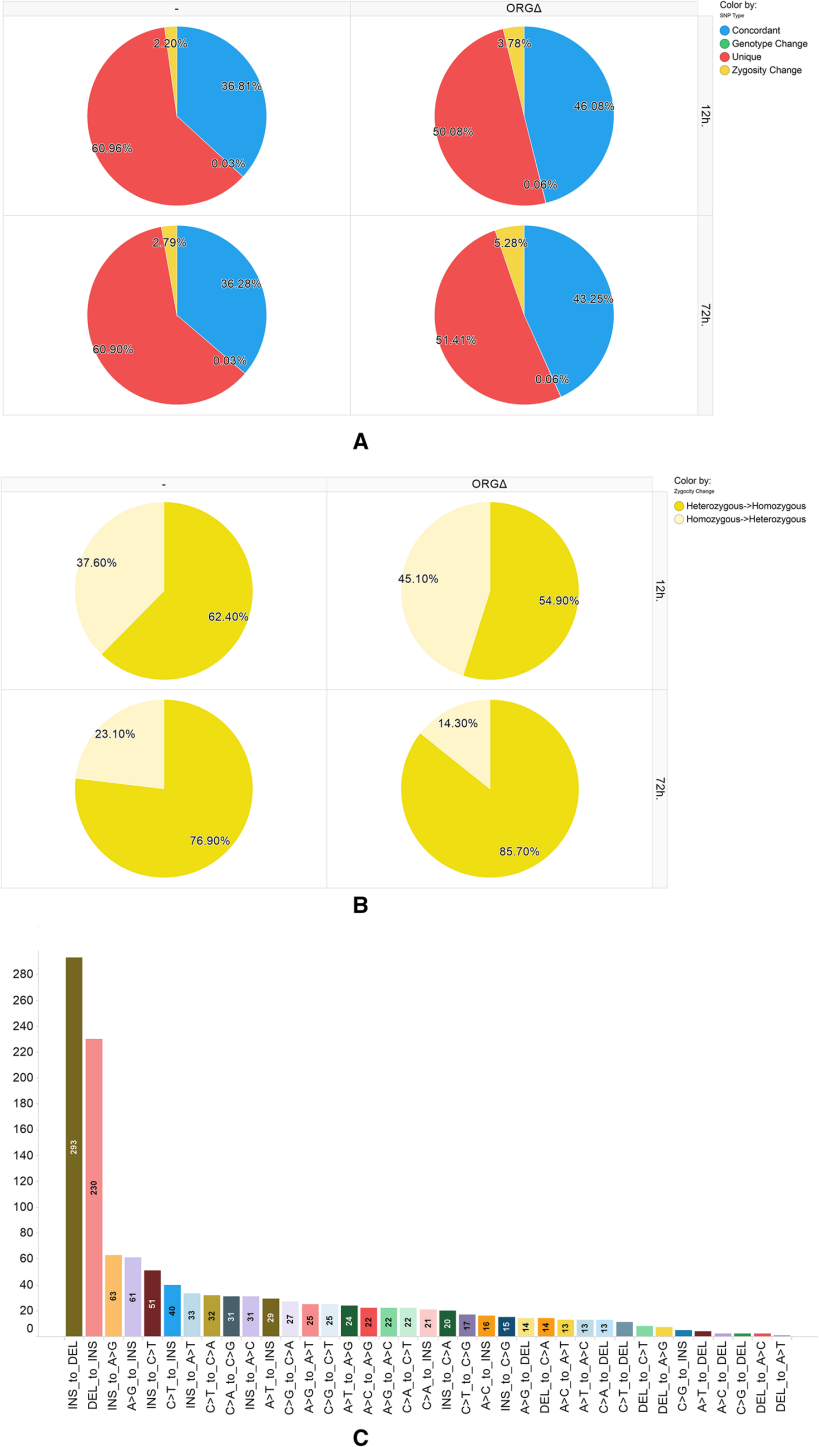
Percentage of SNPs in DNA from 12-h or 72-h formalin-fixed paraffin-embedded (FFPE) tissue samples with or without organocatalyst (ORG∆) treatment relative to paired frozen (FR) DNA samples, classified according to type of change. (**A**) Percent of SNPs identified in FFPE (left) or ORG∆ FFPE (right) relative to matched FR DNA and classified as unique (present in FFPE and not FR), concordant (present in FR and FFPE), zygosity change (predicted to be a homozygous SNP in FFPE but heterozygous in FR or vice versa), and genotype change (predicted to be one genotype in FFPE but a different genotype in FR). (**B**) SNPs identified in FFPE (left) or ORG∆ FFPE (right) relative to matched FR DNA and sub-classified by zygosity change as heterozygous to homozygous (hetero_to_homo) or homozygous to heterozygous (homo_to_hetero). Zygosity changes are FFPE relative to FR. (**C**) Genotype changes between FFPE DNA SNPs compared to FR DNA SNPs. Genotype changes are classified according to their specific genotypes (*INS* insertion, *DEL* deletion).

The types of apparent SNP alteration between preservation groups were also examined. In all comparisons, most SNPs were either completely concordant between a FFPE preservation group and the patient-matched OCT FR samples or were only seen in one group (unique SNPs) (Fig. 2A, Supplemental Files). The SNPs classified as only seen in one group had significantly lower average GQ scores than those see in the concordant SNPs (Table 2). Despite the large similarities in SNP calls between FFPE and FR, ORGΔ treatment had a modest impact on the SNP call breakdown in DNA. For instance, 12-h FFPE DNA samples had 36.8% concordant SNP calls with patient-matched OCT FR while ORGΔ treatment of 12-h FFPE DNA samples increased the percentage of concordant SNP calls with patient-matched OCT FR samples to 46.1%. A similar effect was seen with 72-h FFPE DNA samples in which ORGΔ treatment increased the percentage of concordant SNP calls with patient-matched OCT FR samples from 36.3 to 43.2%. After removing the discordant and concordant SNPs, the remaining SNPs were classified as either zygosity changes (i.e., where a SNP was called heterozygous in one sample and homozygous in the comparative sample) and genotype changes (i.e., where the bases reported between the samples differed). The percent of all SNPs which were classified as zygosity changes increased from 2.2 to 3.78% and from 2.8 to 5.3% in 12-h and 72-h FFPE samples, respectively (Fig. 2A). In all cases, ORGΔ showed an apparent bias towards heterozygous-to-homozygous SNP calls rather than homozygous-to-heterozygous calls (Fig. 2B). This may reflect a strand bias for one strand or the other during purification and amplification of the ORGΔ treatment. The genotype change SNPs also showed an apparent increase from 0.03 to 0.06% in both the 12-h and 72-h FFPE samples (Fig. 2A). Closer examination of the genotype change SNPs shows that most of these changes are insertion/deletion-related (Fig. 2C). The next most common genotype changes were T to A and A to G.

**Table 2:** Average genotype quality scores for FFPE samples.

	FFPE DNA (no ORG∆)	FFPE DNA (ORG∆)	FFPE RNA (ORG∆)
Concordant	279.44 (± 532.35)	384.93 (± 698.60)	37.94 (± 109.43)
Unique	12.34 (± 22.96)	48.39 (± 226.31)	22.82 (± 33.12)

Genotype quality scores for FFPE samples were averaged for SNPs marked as in agreement with the corresponding frozen sample SNPs (concordant) or unique to the FFPE samples. Standard deviation values are given in parentheses. ORG∆ indicates organocatalyst-treated samples. FFPE data were combined from 12-h kidney FFPE, 12-h ovary FFPE, and 72-h ovary FFPE samples.

## Discussion

Damage to nucleic acids during formalin fixation leads to latent sequencing artifacts, which may confound interpretation of genomic data from FFPE samples. In this study, we evaluated effects of organocatalyst treatment during isolation of FFPE DNA on Exome-seq data quality, using paired FFPE and FR human cancer specimens from the NCI BPV program. Use of ORGΔ improved yields for FFPE RNA but not DNA, suggesting differences in optimal incubation conditions based on type of nucleic acid. There were more SNPs identified in DNA from FFPE *vs.* FR compared to SNPs relative to RNA from FFPE *vs.* FR, and the quality of those calls was poorer for RNA, even with equivalent numbers of input reads. More intriguing, ORGΔ treatment improved confidence in SNP calls for all FFPE DNA samples to better than matched FR, which was not the case for ORGΔ FFPE RNA. In general, the concordant SNPs identified in paired FR and FFPE DNA samples agreed on both the genotype and the homozygosity *vs*. heterozygosity of calls regardless of ORGΔ treatment, indicating that DNA is less labile than RNA isolated from FFPE. Treatment with ORGΔ modestly improved concurrent SNP calls in FFPE DNA compared to non-ORGΔ-treated FFPE samples. The increased confidence/quality in ORGΔ FFPE DNA variant calls relative to the matched FR DNA suggests a novel application of ORGΔ treatment, which may improve routine DNA-seq of FR as well as FFPE tissue samples.

In general, it was more challenging to isolate sufficient DNA (> 100 ng) for sequencing from ORGΔ FFPE tissues samples compared to RNA, an issue that has been reported elsewhere for typical (non-ORG treated) FFPE tissue samples^8^. Higher incubation temperatures at 70 °C produced a modest decrease (1.2-fold) in DNA yield for 12-h ORGΔ FFPE ovary samples but a 9.8-fold increase in 72-h ORGΔ FFPE samples, suggesting potential enhancement for lower-quality samples with longer fixation times. However, these results were from a very small sample size and may not be representative or could be a result of variations in nucleic acid isolations. Incubation at 70 °C with ORGΔ also tended to improve insert size between ORGΔ FFPE DNA-seq paired reads for the 12-h *vs.* 72-h samples, but additional work would be needed to confirm this observation. Reasons for the difference in optimal incubation temperatures with ORGΔ may stem from differences in the strength of formaldehyde crosslinks in DNA *vs*. RNA from FFPE tissue samples. Traditional methods of isolating DNA from FFPE tissue samples requires heating at higher temperatures for longer periods. For example, the Qiagen AllPrep kit recommends 90 °C for 2-h to release DNA from formaldehyde crosslinks and 80 °C for 15 min for RNA. The initial incubation temperature optimized for isolation of FFPE RNA with ORGΔ (55 °C) was thus likely suboptimal for DNA. While use of ORGΔ did not appear to consistently improve yields of DNA from FFPE samples, it was not until we increased the incubation temperature to 70 °C that we obtained enough DNA from those samples for Exome sequencing. Therefore, with temperature optimization, ORGΔ may provide additional benefits to sequencing DNA from FFPE tissues samples.

Examination of the SNP GQ scores from the various samples revealed a significant shift toward more SNPs of poor quality in FFPE samples. In the ovary samples at least, this shift toward poorer quality was exacerbated with longer time in formalin. We were unable to examine the effect of time in FFPE with kidney, as 72-h samples were unavailable. Treatment of FFPE samples with ORGΔ resulted in a significant improvement of SNP GQ. Interestingly, the GQ scores in the ORGΔ FFPE DNA samples appeared to be better than those scores from the paired FR samples. A potential explanation for this is that even in the absence of formalin, DNA undergoes natural aldehyde adduct formation^9,10^. Evidence from Ref.^9^ suggests the ORGΔ will indiscriminately reverse aldehyde adducts and cross-linkages on nucleic acid^6^; therefore, we speculate that its use may also reverse natural adducts in DNA from FR samples. Future work is needed to investigate whether ORGΔ treatment can improve SNP detection from FR samples.

The predominant variant classes across preservation groups were unique SNPs and concordant SNPs, accounting for ~ 95% of all SNPs identified. Around 60% of the total SNPs identified in the FFPE DNA samples were unique whereas ~ 36% of SNPs were shared with OCT FR. Treatment of FFPE DNA with ORGΔ resulted in a significant increase in the percentage of concordant SNPs with OCT FR and a corresponding decrease in the percentage of unique SNPs in FFPE tissues samples. For RNA, the number of unique SNPs was markedly higher in OCT FR and even more so in ORGΔ FFPE tissue, compared to FR DNA. Closer examination of the average genotype scores for concordant *vs.* unique SNPs reveals that the unique SNPs were generally low quality in FFPE DNA and RNA. While treatment of FFPE DNA with the ORGΔ more than tripled the average genotype score for the unique SNPs, the average was still well below the average score for SNPs concordant with FR. The major difference observed in DNA variants from FFPE tissue samples compared to matched FR were apparent changes in heterozygosity, which may reflect a bias toward one chromosome of a pair during amplification due to irreversible damage or fragmentation. This finding was further supported by the increased proportion of heterozygous to homozygous changes observed in the 72-h FFPE samples. Treatment with ORGΔ partially reversed that trend, suggesting that it may be partially reversing fixation-related damage in DNA isolated from FFPE tissue samples, as previously shown for FFPE RNA^5,6^ of the millions of SNPs examined, less than 0.1% were apparent genotype changes. Of these changes, the majority were insertion-to-deletion and deletion-to-insertion changes.

Previous research reported high concordance (> 99%) in sequence variations between FFPE RNA and exonic DNA from corresponding FR tumor samples^2^. In the current study, incorporating the ORGΔ FFPE RNA data into our analysis added little value in enhancing confidence of FFPE DNA SNP calls. Directly comparing our results to Graw et al*.*^2^ is challenging due to inherent methodologic differences. Graw et al*.*^2^ compared paired FR *vs*. FFPE tumor samples to identify differences in genotype, while we compared each sample to a reference list of SNPs followed by comparisons between paired FR *vs*. preservation samples. Despite these differences, when focusing on genotype changes, which is the most valid comparison between the two studies, we both identified a few hundred SNP differences between FFPE DNA and FR, suggesting modest benefit of RNA-seq data for recently collected FFPE samples with relatively short fixation times. We also both identified a shift toward homozygous calls in RNA and DNA FFPE. Similar to Graw et al*.*^2^*,* another study^11^, reported that combining RNA-seq and DNA Exome-seq enhanced detection of variants and identification of mutations in tumors over DNA Exome-seq alone. One major difference between this study and ours is this study was performed using data from fresh frozen tumor/normal paired tissue samples, not FR and FFPE tumor only paired tissue samples. Despite this, we observed that combining RNA-seq data with Exome-seq data resulted in generally higher genotype quality scores for many of the SNPS identified by DNA Exome-seq data alone like^11^. This likely reflects the greater coverage of a number of SNPs when the RNA-seq reads are added to the analysis. Because we lacked tumor/normal tissue pairs, we could not evaluate the utility of this approach for enhancing detection of mutation frequencies on our samples.

Other groups have tried alternative methodologies to remove aberrant reads from FFPE samples prior to analysis. For example, Wei et al*.*^12^ used a bioinformatic approach to remove chimeric reads prior to identification of structural variants, whereas Haile et al*.*^13^ used an S1 nuclease step to remove single-stranded fragments and overhangs and thereby reduce chimeric strand-split artifact reads. In contrast, we chose to use stringent genomic alignment parameters to remove most of the artifactual chimeric reads. Future work should focus on directly comparing and integrating available experimental (e.g. ORGΔ and S1 nuclease) and bioinformatic methods to improve variant identification and accuracy in FFPE samples.

With the increasing emphasis on precision medicine approaches in patient care and integration of genome-based information in diagnosis and treatment of disease, there is corresponding need for enhanced next generation sequencing approaches to FFPE tissue specimens. DNA extracted from formalin-fixed tissues is highly fragmented and replete in crosslinks and other lesions, which may induce artifacts during sequencing that are difficult to distinguish from actual mutations. Tumor heterogeneity may introduce additional complexity and further complicate interpretation of sequencing data. Technically, there is a major need to “demodify” DNA from FFPE samples and enable more accurate identification of mutations. The work described here adds to our understanding of sequence artifacts and strategies to improve variant detection in FFPE tissues. The organocatalyst method, once optimized for DNA, may increase confidence in variant detection and enhance use of valuable clinical specimens.

## Materials and methods

### Experimental overview

**Figure 3:**
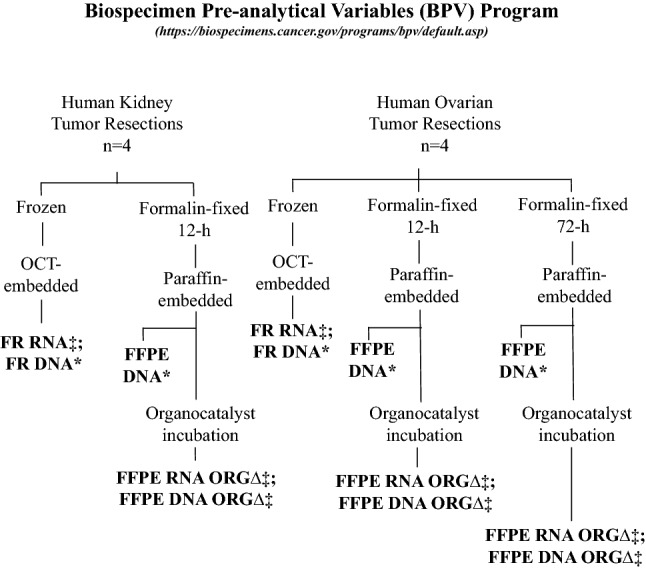
Experimental design with sample types and procedures performed to evaluate the effects of organocatalyst (ORG∆) treatment on DNA isolated from formalin-fixed paraffin-embedded (FFPE) tissue samples. All ORG∆ groups used an 18-h incubation at 55 °C unless otherwise indicated (see Supplementary Table 2). *Previously sequenced at Q2 Solutions (by NCI). ^‡^Sequenced at Q2 Solutions (by EPA).

The experimental design is presented in Fig. 3. Briefly, human primary renal and ovarian carcinoma specimens were obtained by the NCI BPV program following an institutional review board–approved protocol^7^. These tissues were collected under prospectively designed BPV program conditions to evaluate common pre-analytical variables that may impact molecular profiling of preserved biospecimens (https://biospecimens.cancer.gov/programs/bpv/). Variables investigated in the present study included sample preservation methods (i.e., freezing *vs*. formalin fixation) and time in formalin.

Samples were originally selected from a list of 243 total frozen and FFPE samples available from the BPV program. These samples were derived from 37 unique cancer cases covering four types of cancer: serous carcinoma of the ovary, clear cell renal carcinoma, adenocarcinoma of the colon, and serous carcinoma of the fallopian tube. Each specimen had been divided and processed according to different BPV protocols (see https://biospecimens.cancer.gov/programs/bpv/default.asp and https://brd.nci.nih.gov/brd/sop-compendium/show/1181 for background information). Protocols A-D varied by time in fixative (6, 12, 23, and 72-h, respectively), whereas Protocols E–H varied by delay to fixation (1, 2, 3, and 12-h, respectively). Not all protocols were represented for each cancer specimen.

For our analysis, we selected Protocol B (12-h fix) to represent a standard “short-fix” scenario and Protocol D (72-h fix) to represent a standard “long-fix” scenario. Criteria for sample selection included the availability of 4 samples per protocol with corresponding frozen samples and DNA-Seq data (without ORGΔ)^4^. There were not sufficient colon or fallopian tube cancer cases to meet these criteria; therefore, we focused on available ovary (n = 4) and kidney (n = 5) cancer cases. Among the five kidney cases, four were arbitrarily selected to analyze.

Paired kidney tumor resections arrived at the repository between 2013 and 2014 and were either frozen and embedded in optimal cutting temperature compound (OCT) (FSC22 Clear, Leica Biosystems, Buffalo, IL) or fixed in 10% neutral buffered formalin (NBF) for 12-h and embedded in paraffin. (Note that many archived specimens are fixed in formalin for prolonged times, often ≥ 24-h). Tumor sections of ovary were prepared the same as for kidney with an additional time point after 72-h of formalin fixation.

All tissue samples were sectioned at the Van Andel Research Institute (VARI) (Grand Rapids, MI), where NCI biospecimens are stored. The presence of tumor tissue was microscopically confirmed by a certified pathologist in tissue sections stained with hematoxylin and eosin (H&E) directly adjacent to those used for RNA and DNA isolations. The H&E-stained sections were also assessed for cellularity using image analysis of scanned images from an Aperio AT2 Slide Scanner (Leica Biosystems, Buffalo Grove, IL) (Supplementary Table 6). DNA isolation from FR ovary and kidney sections and their matched FFPE sections was performed by NCI^7^. DNA isolation with ORGΔ from FFPE samples and RNA isolation from OCT-embedded FR and FFPE samples with or without ORGΔ were performed on matched samples by the U.S. Environmental Protection Agency (EPA) (Research Triangle Park, NC).

### Human subjects

Human tumor specimens used in this study were collected at four medical centers using Institutional Review Board (IRB)-approved protocol for the collection of human biospecimens for research purposes in accordance with the Helsinki Declaration of 1975, as revised in 1983 (Emory University IRB00045796 [approved March 21, 2013]; University of New Mexico IRB00000591 [approved June 28, 2012]; University of Pittsburgh IRB0106147 [approved May 28, 2014], IRB0411047 [approved July 18, 2014], IRB09502110, IRB0506140 [approved May 28, 2014], and IRB056140 [approved June 19, 2014]; and Boston Medical Center IRB00000376 [approved February 05, 2014]). Biospecimens were only collected from patients who met pre-surgery inclusion criteria and provided written informed consent^7^.

### RNA and DNA isolation

Total RNA was isolated from three 10-µm-thick sections of FR OCT-embedded ovary or kidney tumor samples, as previously described^14^. Tissue sections were immersed in 600 µL of RNAzolRT (Molecular Research Center, Cincinnati, OH), vortexed, and then purified by the RNeasy MinElute column according to manufacturer recommendations (Qiagen GmbH, Hilden, Germany). For FFPE samples, total RNA was isolated from three to six 10-µm-thick paraffin sections, while DNA was isolated from six to ten 10-µm-thick paraffin sections. Tissue sectioning was performed at VARI under RNase- and DNase-free conditions. Samples were approximately 3–4 years of age in block. Sections were collected and sealed in sterile RNase- and DNase-free microcentrifuge tubes before overnight shipment to the U.S. EPA on dry ice. Once received, FFPE tissue curls were stored at − 80 °C prior to nucleic acid isolation. For total RNA isolations, FFPE sections were deparaffinized (Qiagen Deparaffinization Solution, catalog no. 19093), digested with proteinase K, and incubated for ~ 18 h at 55 °C with 40 mM of NaOH-buffered 2-amino-5-methylphenyl phosphonic acid organocatalyst (final concentration 20 mM, pH 7.0; Evans Analytical Group, Maryland Heights, MO) using the Qiagen AllPrep^®^ DNA/RNA kit (catalog no. 80234; Qiagen), as described in Wehmas et al.^5^. The ORGΔ process decreases formaldehyde-induced aminal cross-links and hemi-aminal adducts on nucleic acids^6^.

For DNA isolation, FFPE sections were deparaffinized (Qiagen Deparaffinization Solution, catalog no. 19093) and digested with proteinase K for 15 min. Following removal of the RNA-containing supernatant, the DNA pellet was digested for an additional 60 min in proteinase K, as directed in the manufacturer protocol; however, instead of a 120-min incubation at 90 °C, the DNA was incubated for ~ 18-h at 55 °C with 40 mM of the organocatalyst (final concentration 20 mM, pH 7.0) and purified using the Qiagen AllPrep^®^ DNA/RNA kit (catalog no. 80234; Qiagen). Due to challenges in obtaining enough DNA for Exome-seq, the incubation temperature with ORGΔ was increased to 70 °C for a subset of the tissue samples (Supplementary Table 7). Refer to Supplementary Information for a detailed protocol on the RNA and DNA isolation from FFPE tissue samples. The concentration of RNA and DNA obtained from all samples was measured using a Qubit 2.0 fluorometer (Invitrogen, Carlsbad, CA); final amounts submitted for sequencing are included in Supplementary Tables 7 and 8. RNA integrity was evaluated by an Agilent 2100 Bioanalyzer (Agilent Technologies GmbH, Berlin, Germany) (Supplementary Table 8). All RNA samples were stored at − 80 °C and all DNA samples were stored at − 20 °C until sequencing.

### RNA sequencing and analysis

RNA library preparation and sequencing were completed at Expression Analysis (10/2018, EA Genomic Services, Q2 Solutions—a Quintiles Quest Joint Venture, Durham, NC), as described previously^14^. FFPE RNA underwent reduced (or no) fragmentation during library preparation, depending on Agilent Bioanalyzer profiles. RNA was ribo-depleted and cDNA libraries were synthesized using the TruSeq Stranded Total RNA Library Prep Kit with Ribo-Zero (catalog no. RS-122-2303; Illumina, San Diego, CA). Paired-end 50 base pair sequencing to at least 25 million reads per sample was performed on Illumina HiSeq 2500 instruments. Mean sequencing depth was 29.2 to 30.0 million reads within each tissue and preservation group with an average read Phred score of 35.9 to 36.2 per tissue and preservation group (Supplementary Table 9). Base call files were transformed into FASTQ files via bcl2fastq version 2.20 (Illumina). See Supplementary Table 9 for RNA pre-alignment quality metrics by individual sample.

### DNA sequencing and analysis

DNA library preparation and sequencing were also run at Expression Analysis (10/2018). Genomic FFPE DNA underwent reduced (or no) fragmentation during library preparation, depending on Agilent Bioanalyzer or TapeStation profiles. DNA libraries were prepared using Agilent SureSelect chemistry followed by hybridization using probes specific for the exonic region of the genome (ExomeSeq version 5). Captured DNA libraries were then amplified and indexed for Illumina sequencing. Generated DNA libraries were quantified by qPCR using primers specific for Illumina-sequencing adapters. Qualitative analysis was performed using Agilent TapeStation or equivalent technology. Concentration- and size-normalized DNA libraries were analyzed using clonal single molecule array technology, followed by sequencing with synthesis technology using reversible terminator chemistry to a targeted exonic read depth of 45 million (~ 100× coverage) using 100 base pair, paired-end sequencing. Actual sequencing depth ranged from 59.4 to 73.5 million reads with Phred scores between 35.5 and 36.4 per tissue and preservation group on average (Supplementary Table 10). Base call files were transformed into FASTQ using bcl2fastq. See Supplementary Table 10 for DNA pre-alignment data by individual sample.

Statistical tests of RNA and DNA pre-sequencing and pre-alignment data were conducted to determine preservation-related differences between matched FR and FFPE samples using the *stats* and *car* packages within R (R Core Team 2019, version 3.5.0; https://www.R-project.org/). Data were initially screened for normality and homogeneity of variance using Shapiro–Wilk’s Test and Levene’s Test, respectively. Due to small samples sizes and lack of normality across many metrics, nonparametric statistical methods were employed. Data from kidney samples were analyzed using a paired Wilcoxon Signed-Rank Test (two-tailed, p-value < 0.05). Unlike kidney samples, ovary had an additional level of comparison (FR, 12-h ORGΔ FFPE, and 72-h ORGΔ FFPE); therefore, group level differences were first assessed by Friedman test. Groups with significant preservation-related effects (p-value < 0.05) were then analyzed by paired Wilcoxon Signed-Rank Test (two-tailed, Holm-adjusted p-value < 0.05).

### SNP analysis

To ensure that DNA and RNA SNP calls were generated equivalently, all reads were aligned to the reference chromosome using STAR version 2.5.2a. (Note: Reads were not trimmed prior to alignment as GATK tools account for base qualities. For more information, see https://gatkforums.broadinstitute.org/gatk/discussion/2957/read-trimming.) Alignments were performed using default parameters, except the following: –outSAMmapqUnique 60 –alignSJDBoverhangMin 1 –outFilterMismatchNoverLmax 0.05 –outFilterScoreMinOverLread 0.90 –outFilterMatchNminOverLread 0.90 –alignIntronMax 1000000. All reads were aligned to the human genome version GRCh38.

SNPs were identified using GATK version 4.0.10.1 with a modified version of the workflow recommended for Germline short variant discovery through the HaplotypeCaller step. The 151 build of dbSNP (reference = GRCh38.p7; fileDate = 20180418) was used for SNP identification (ftp://ftp.ncbi.nih.gov/snp/organisms/human_9606/VCF/All_20180418.vcf.gz; accessed Oct 23, 2018). Quality of the assigned genotype (GQ) for each SNP was calculated as described in the GATK documentation (https://gatk.broadinstitute.org/hc/en-us/articles/360035890451-Calculation-of-PL-and-GQ-by-HaplotypeCaller-and-GenotypeGVCFs; last accessed Dec 18, 2020) except that the scores were not capped at 99. GQ scores for the different treatment groups were compared by plotting GQ score *vs*. cumulative percent of all SNPs for a given group with GQ scores at or below that value. Resulting curves were analyzed using the Kolmogorov–Smirnov test (ks.test) within R. For the analyses, GQ scores above 300 (~ 90% of all DNA FR sample scores were at or below this value) were not included. SNP zygosity and genotype were also extracted from the GATK-generated variant calls for each sample. Both zygosity and genotypes were compared between samples from the same individual (subjected to different treatment and isolation protocols).

SNPs were classified as: (1) concordant (SNP calls were identical between sample preparations), (2) unique (SNPs that were called in one sample preparation but not in the comparator sample), (3) zygosity change (SNPs that were identified as heterozygous in one sample preparation but homozygous in the comparator preparation regardless of genotype change), and (4) genotype change (SNPs for which there was a difference in genotype change between the two preparations). SNP counts from each of the four classes were then normalized by dividing by the total number of SNPs for each sample type.

### Ethical approval

The authors confirm that all methods were carried out in accordance with relevant guidelines and regulations.

## Supplementary Information

Supplementary Legends.

Supplementary Figure 1.

Supplementary Figure 2.

Supplementary Tables.

Supplementary Information.
