# Supplementary material for: Organocatalyst treatment improves variant calling and mutant detection in archival clinical samples
Source: Sci Rep. 2022 Apr 20;12:6509. doi: 10.1038/s41598-022-10301-0 (PMC9021284; doi:10.1038/s41598-022-10301-0)
Supplement: Supplementary file 1 — Supplementary Legends. [file 41598_2022_10301_MOESM1_ESM.docx]

**Organocatalyst treatment improves variant calling and mutant detection in archival clinical samples**

Leah C. Wehmas*^§^, Charles E. Wood*^, Ping Guan^¥^, Mark Gosink^±^, Susan D. Hester^*^

*Office of Research and Development, U.S. Environmental Protection Agency, Research Triangle Park, NC

^Current affiliation: Boehringer Ingelheim Pharmaceuticals, Inc., Ridgefield, CT

^¥^National Cancer Institute, Bethesda, MD

^±^Pfizer, Groton, CT

The author(s) declare no competing interests. Note: One coauthor is an employee of Pfizer.

^§^To whom correspondence should be addressed: MD-B105-03, 109 T.W. Alexander Drive, U.S. Environmental Protection Agency, Research Triangle Park, NC, 27709 USA; Email: [wehmas.leah@epa.gov](mailto:wehmas.leah@epa.gov). ORCID #0000-0003-1529-2387

**Key words:** DNA, FFPE, sequencing, variant, SNP, formalin, biorepository, archival samples

**SUPPLEMENTARY MATERIAL**

**Supplementary Figure 1.** Total RNA isolated from tissues that were either frozen (FR) or fixed in formalin for 12-h. or 72-h. prior to embedding in paraffin (FFPE) or total DNA isolated from 12-h. or 72-h. FFPE. Note, DNA was not isolated from FR samples in the present study as these samples already had exome sequencing information from the BPV program. Therefore, this data is not included in the lower two panels. The amount of nucleic acid was normalized to the approximate cellularity, as measured in adjacent tissue sections. The unshaded boxes in the upper two panels represent RNA isolated from FR tissue using standard procedures. Gray boxes represent DNA and RNA isolated from FFPE tissue using organocatalyst (ORG∆) with incubation at 55°C. Minimal to no DNA was obtained from 72-h FFPE ovary tumor resections that underwent isolation using ORG∆ with incubation at 55°C, which is represented by a dash at 0. Red boxes represent DNA isolated from FFPE tissue using ORG∆ with incubation at 70°C.

**Supplementary Figure 2**. Influence of organocatalyst (ORG∆) treatment on cumulative percent SNP genotype quality (GQ) scores by tissue. GQ scores for the indicated sets of samples were extracted from the VCF files generated by GATK. The percent of SNPs for each sample type scoring at or below the indicated scores on the x-axis are displayed on the y-axis. SNP GQ scores from DNA samples that had been frozen (FR) or formalin-fixed (FFPE) for 12-h. with and without ORG∆.

**Supplementary Table 1.** Summary statistics on effects of formalin fixation on RNA quality and quantity

**Supplementary Table 2.** Summary of the quantity of nucleic acid per cell isolated from matched tissue samples by ORG incubation temperature

**Supplementary Table 3.** Summary of pre-alignment RNA sequencing metrics for frozen, ORG 12 and ORG 72 h. formalin -fixed paraffin-embedded tissue samples

**Supplementary Table 4.** Summary of pre-alignment DNA exome sequencing metrics for ORG 12 and 72 h. formalin -fixed paraffin-embedded tissue samples

**Supplementary Table 5.** Pre-alignment DNA exome sequencing metrics for ORG 12 and 72 h. formalin -fixed paraffin-embedded tissue samples

**Supplementary Table 6.** Summary of cell count metrics from adjacent microtome section of tumor tissue

**Supplementary Table 8.** Quantity of DNA isolated from 12 h. and 72 h. ORG FFPE

Supplementary Table 7. RNA quality and quantity from OCT (Frozen) preserved, 12 h. formalin-fixed, and 72 h. formalin-fixed paraffin-embedded tumor resections.

**Supplementary Table 9.** Pre-alignment RNA sequencing metrics for frozen (FR), ORG 12 and ORG 72 h. formalin-fixed paraffin-embedded (FFPE) tissue samples

**Supplementary Table 10.** Pre-alignment DNA exome sequencing metrics for ORG 12 and 72 h. formalin -fixed paraffin-embedded tissue samples
