# Supplementary material for: Organocatalyst treatment improves variant calling and mutant detection in archival clinical samples
Source: Sci Rep. 2022 Apr 20;12:6509. doi: 10.1038/s41598-022-10301-0 (PMC9021284; doi:10.1038/s41598-022-10301-0)
Supplement: Supplementary file 2 — Supplementary Figure 1. [file 41598_2022_10301_MOESM2_ESM.pdf]

Organocatalyst treatment improves variant calling and mutant detection in archival clinical samples .Leah C. Wehmas*§, Charles E. Wood*^, Ping Guan¥, Mark Gosink±, Susan D. Hester*

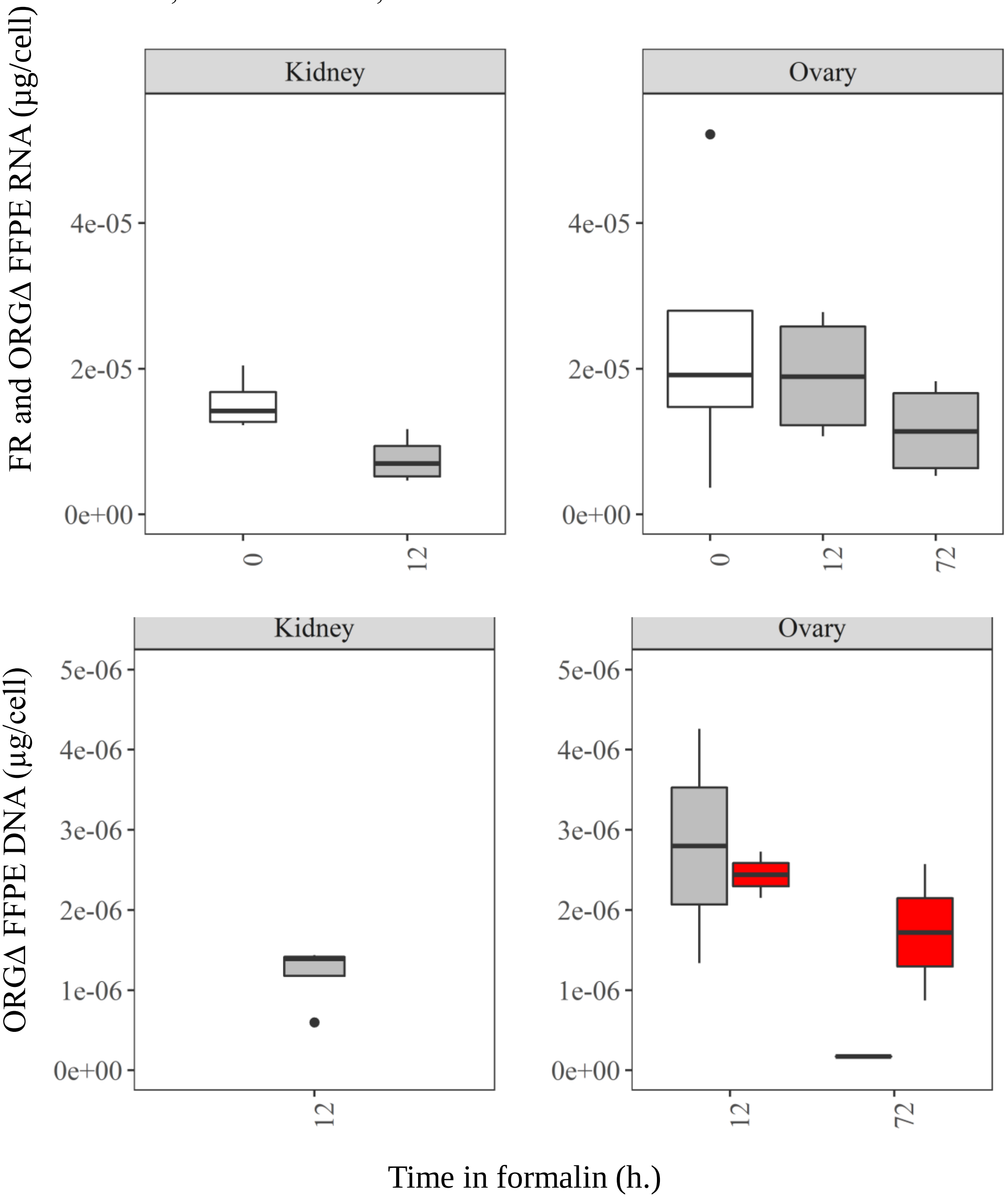

Supplementary Figure S1.
