# Supplementary figures and images for: Organocatalyst treatment improves variant calling and mutant detection in archival clinical samples
Source: Sci Rep. 2022 Apr 20;12:6509. doi: 10.1038/s41598-022-10301-0 (PMC9021284; doi:10.1038/s41598-022-10301-0)

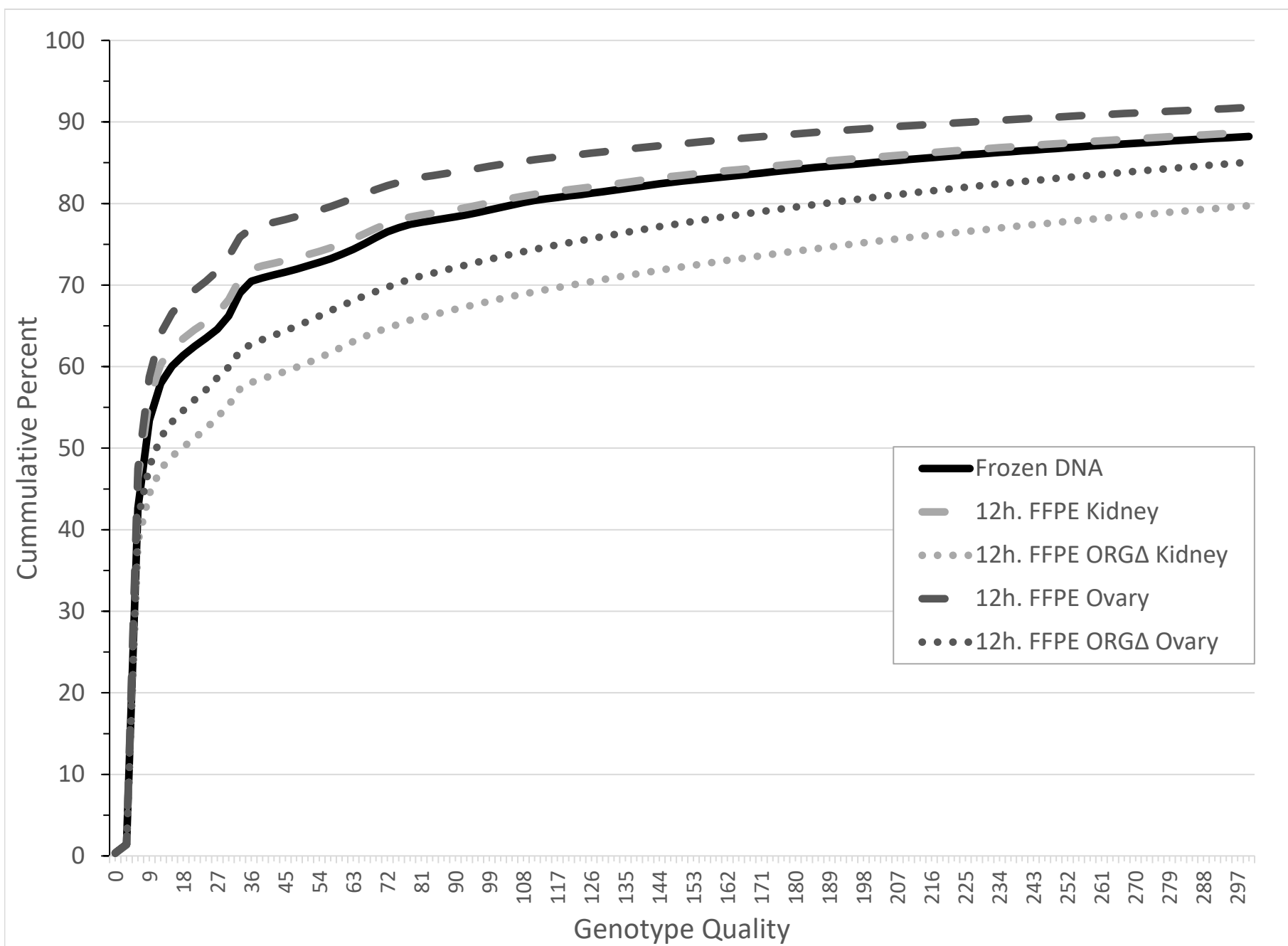

Supplement: Supplementary file 3 — Supplementary Figure 2. [file 41598_2022_10301_MOESM3_ESM.pdf]
