# Supplementary material for: Organocatalyst treatment improves variant calling and mutant detection in archival clinical samples
Source: Sci Rep. 2022 Apr 20;12:6509. doi: 10.1038/s41598-022-10301-0 (PMC9021284; doi:10.1038/s41598-022-10301-0)
Supplement: Supplementary file 5 — Supplementary Information. [file 41598_2022_10301_MOESM5_ESM.docx]

**Organocatalyst treatment improves variant calling and mutant detection in archival clinical samples**

Leah C. Wehmas*^§^, Charles E. Wood*^, Ping Guan^¥^, Mark Gosink^±^, Susan D. Hester^*^

*Office of Research and Development, U.S. Environmental Protection Agency, Research Triangle Park, NC

^Current affiliation: Boehringer Ingelheim Pharmaceuticals, Inc., Ridgefield, CT

^¥^National Cancer Institute, Bethesda, MD

^±^Pfizer, Groton, CT

The author(s) declare no competing interests. Note: One coauthor is an employee of Pfizer.

^§^To whom correspondence should be addressed: MD-B105-03, 109 T.W. Alexander Drive, U.S. Environmental Protection Agency, Research Triangle Park, NC, 27709 USA; Email: [wehmas.leah@epa.gov](mailto:wehmas.leah@epa.gov). ORCID #0000-0003-1529-2387

**Key words:** DNA, FFPE, sequencing, variant, SNP, formalin, biorepository, archival samples

**Supplementary Information**

**OPERATING PROCEDURE**

**Enhanced Isolation of DNA/RNA from Formalin-fixed, Paraffin-embedded Samples: Modified Qiagen Protocol**

1. **SCOPE OF APPLICATION (LIMITATIONS)**

The purpose of this OP is to provide an optimized extraction and cleanup technique for DNA/RNA from formalin-fixed, paraffin-embedded tissue (FFPE) specimens. FFPE samples present challenges for genomic analysis because of formaldehyde-induced adducts, crosslinks, and other modifications to FFPE DNA/RNA, which can interfere with RNA-sequencing and other methods for measuring gene expression. Commercially-available products targeted to FFPE specimens use buffers and heat to reverse adducts and enable extraction of nucleotides. Recent literature demonstrates that aminal nucleotide adducts, in particular aminal dimers, are resistant to this process, and the temperatures used are often damaging to the integrity of both RNA and DNA. The protocol described here is designed to enhance the extraction and cleanup of nucleotides from FFPE specimens by addressing the issues of adduct reversal and potential heat-induced hydrolysis. It is based on the protocol and use of the Qiagen AllPrep^®^ DNA/RNA FFPE kit, with additional modifications as proposed in the literature (Evers *et al.*, 2011; Karmakar *et al.*, 2015). These modifications utilize a water-soluble catalyst to facilitate the reversal of formaldehyde nucleic acid adducts, enhancing both yield and quality of DNA and RNA extracts from the Qiagen protocol.

**2. PREREQUISITES**

**2.1 EQUIPMENT AND SUPPLIES**

Equipment

Chemical Fume Hood

Benchtop Micro-centrifuge, 1.5-2mL tube capacity

Benchtop vortex mixer

Sonication bath

Heat blocks, water bath or heated orbital incubator for microfuge tubes, 50 to 90˚C range capacity

Laboratory glassware (small volume)

Consumables and Supplies

Qiagen AllPrep® DNA/RNA FFPE kit (Qiagen cat #80234)

Deparaffinization Solution (Qiagen cat #19093)

RNAse A, 100mg/mL

RNaseZAP^®^ (Thermo Fisher Scientific) or similar RNase decontamination product

Nuclease free water

96-100% Ethanol, molecular biology grade

96-100% Isopropanol, molecular biology grade

NaOH solution, 0.1 N

(2-amino-5-methylphenyl)phosphonic acid, lyophilized powder

Sterile, RNAse-free pipette tips

Sterile nuclease-free microfuge tubes, 1.5 and 2.0mL

Amber Glass sample vials with lids, 8mL

Glass stirring rod

Hydrion S/R pH paper, range 4.5 to 8.5

**2.2 TRAINING REQUIREMENTS**

Laboratory Safety Core Classes

Basic Molecular Biology Techniques

Basic Organic Chemistry/Biochemistry Techniques

**3. CAUTIONARY NOTES OR SPECIAL CONSIDERATIONS**

Particular note should be made of the recommendations regarding starting material as outlined in the Qiagen product manual under *Important Notes* (Qiagen, 2014). At all times, attempts should be made to protect the integrity of the samples and avoid cross-contamination. All steps in this extraction are to be performed as described to ensure optimum yield. Aseptic technique suitable for molecular biology should be employed, including frequent glove changes and cleansing of bench and glove surfaces with an RNAse spray such as RNaseZAP^®^ (Thermo Fisher Scientific). See Appendix A of the Qiagen AllPrep^®^ DNA/RNA FFPE Handbook for additional recommendations on the handling of RNA. Preparation of Catalyst Solution is described in the Appendix of this OP. It contains (2-amino-5-methylphenyl) phosphonic acid; therefore, standard laboratory procedures and precautions for handling and making solutions with organic compounds should be followed at all times (e.g. personal protective equipment, use of hood, etc.).

**4. PROCEDURE**

Ensure all buffers and solutions for the AllPrep^®^ kit have been prepared, stored and handled according to the guidelines in the product manual. All but the DNase I incubation mix can be prepared in advance. Organocatalyst solution should be prepared prior to isolation. Refer to Appendix of this OP for preparation of catalyst solution and a workflow schematic for the isolation protocol.

Deparaffinization and release of nucleotide material from FFPE specimens

1. Cut fresh 10-20µm sections under nuclease-free conditions using microtome. Exclude first section when starting to cut. Depending on surface area of tissue in paraffin block, more sections may be needed to obtain adequate yields.
2. Immediately place sections in 1.5mL sterile, nuclease-free microfuge tube and close cap.
3. Add 640µL deparaffinization solution for typical digest. More deparaffinization solution can be added for increased sample loads (*e.g.* 960µL for 6x10µm sections).
4. Vortex capped microfuge tube for 10sec, centrifuge briefly to immerse sample completely.
5. Incubate at 56˚C for 3 min, cool to room temperature (RT) (15-25˚C) and centrifuge at full speed (e.g. 20,000 x g) for 2-3 min.
6. Remove supernatant without disturbing pellet; use fine pipette tip to maximize removal of deparaffinization solution.
7. Incubate at 37˚C with lid open for 10 min to dry pellet; inspect at halfway point and remove excess solution with fine pipette tip as necessary.
8. Add 150µL Buffer PKD to pellet; flick tube to resuspend in buffer. Add 10µL Proteinase K and vortex.
9. Incubate at 56˚C for 15 min; then cool on ice for 3-4 min. *Complete cooling is important for efficient precipitation.*
10. When sample is completely cooled, centrifuge for 15 min at 20,000 x g.
11. Without disturbing pellet, transfer supernatant to a 2mL sterile, nuclease-free microfuge tube for RNA extraction. *Store DNA-containing pellet in situ at -20˚C for later DNA extraction*.

RNA Isolation

1. To the tube in step 11, immediately add 150µL of 40mM Catalyst solution pH 7.0 (1:1, V/V) to final concentration of 20mM, and incubate for 18 hours at 55˚C.
2. After incubation, cool to RT, briefly centrifuge to remove drops on lid, and add 640µL of Buffer RLT. Mix by pipetting or vortexing.
3. Add 2240µL ethanol (96-100%) and Mix well by vortexing or pipetting.

*Note:* *Precipitants may form, but this will not affect yield.*

1. Transfer 700µL of contents to RNeasy MinElute spin column/collection tube. Centrifuge capped column assembly for 15 sec at 8000+ x g. Discard flow through.

*Note: Centrifugation temperatures below 15˚C can cause precipitants to form and clog the membranes in the RNeasy spin columns.*

1. Repeat step 15 reusing the same column assembly until all of final sample solution from step 14 has passed through the column.
2. Add 350µL of Buffer FRN to the spin column, cap and centrifuge at 8000+ x g for 15 sec. Discard flow through.
3. Prepare DNase I incubation mix using 10µL DNase I stock solution to 70µL Buffer RDD per sample column to be processed. *Mix gently by inversion*.
4. Add 80µL of DNase I incubation mix to surface of spin column membrane, and incubate at RT for 15 min.
5. Add 500µL Buffer FRN to spin column, centrifuge at 8000+ x g for 15 sec. **Save the flow-through for use in step 21.**

*Do not discard the flow-through, as it contains RNA including small RNAs.*

1. Place RNeasy MinElute spin column in new collection tube and apply saved eluent from step 20. Centrifuge at 8000+ x g for 15 sec. (*This flow though can be discarded.*)
2. Wash step 1: Add 500µL Buffer RPE to spin column, cap and centrifuge at 8000+ x g for 15 sec. Discard flow through.
3. Wash step 2: Add 500µL Buffer RPE to spin column, cap and centrifuge at 8000+ x g for 15 sec. Discard flow through.
4. Place RNeasy MinElute spin column in new collection tube and centrifuge at full speed with **open lid** for 5 min. Discard collection tube with flow through.

*Note: Dry spin is essential for removal of residual ethanol which will affect sample yield and integrity in downstream applications*.

1. Place RNeasy MinElute spin column in sterile, nuclease-free microfuge tube. Add 14-30µL RNase-free water to spin column membrane and incubate for 1 minute at RT. Centrifuge at full speed for 2 min.
2. Perform QC for yield and purity using Nanodrop and/or Qubit as appropriate for downstream use.

Isolation of Genomic DNA

1. Bring pellet from step 11 to RT, and resuspend in 180µL ATL Buffer.
2. Add 40L Proteinase K, vortex and incubate for 1 hour at 56˚C.
3. After one hour, add 220µL of 40mM Catalyst solution pH 7.0 (1:1, V/V) to final concentration of 20mM, and incubate for 18 hours at 55˚C or 70˚C.
4. Cool tube to RT and briefly centrifuge to remove drops form inside of the lid.
5. Add 4µL RNase A (100mg/mL) and incubate for 2 min at RT.
6. Add 400µL Buffer AL and vortex.
7. Add 400µL ethanol (96-100%) and vortex again.

*Note: Precipitants may form, but this will not affect yield.*

1. Transfer all of sample to QIAamp MinElute spin column/collection tube assembly. Cap and centrifuge at 8000+ x g for 1 min. Discard flow through.

*Note: If all sample does not pass through column, warm to RT and re-centrifuge sample.* *Centrifugation temperatures below 15˚C can cause precipitants to form and clog the membranes in the QIAamp spin columns.*

1. Place spin column in new collection tube, add 700µL Buffer AW1, and centrifuge at 8000+ x g for 15 sec. Discard flow through.
2. Add 700µL Buffer AW2 to QIAamp MinElute spin column/collection tube assembly. Centrifuge at 8000+ x g for 15 sec. Discard flow through.
3. Add 700µL ethanol (96-100%) to QIAamp MinElute spin column/collection tube assembly. Centrifuge at 8000+ x g for 15 sec. Discard flow through and collection tube.
4. Place the QIAamp MinElute spin column in a new 2 ml collection tube. Open the lid of the spin column, and centrifuge at full speed for 5 min. Discard the collection tube with the flow-through.
5. Place QIAamp MinElute spin column in sterile, nuclease-free microfuge tube. Add 30-100µL Buffer ATE to spin column membrane and incubate for 5 minute at RT. Centrifuge at full speed for 2 min.
6. Perform QC for yield and purity using Nanodrop and/or Qubit
7. Store final eluent at -20˚C for future use.

**5. QUALITY CONTROL RATIONALE**

Nucleic acids purified from FFPE samples do not routinely match yield and quality of snap-frozen or fresh tissues; therefore, strict adherence to the protocol is important for best results. All DNA/RNA isolated from FFPE samples using this protocol should be evaluated for quality using standard metrics (e.g. yield, 260/280, Agilent Bioanalyzer) before use in RNA-sequencing or other analyses. Initial RNA samples isolated using this protocol will be used for RNA-sequencing, and these results will be compared with paired RNA samples isolated using the standard Qiagen AllPrep^®^ protocol. The best method will then be determined based on sequencing quality parameters (clusters, reads, percent alignment, etc.). Future protocols are also being developed to evaluate FFPE RNA quality prior to sequencing. Genomic DNA contamination of the RNA isolates can also be an issue if the tissue is DNA-rich. The Qiagen AllPrep^®^ DNA/RNA FFPE Handbook contains additional resources to assist with addressing DNA contamination and other issues. The evaluation of purity and yield for FFPE samples must be viewed from within the context of the source material and intended use/analysis of the isolates.

**6. REFERENCES**

Evers, D. L., Fowler, C. B., Cunningham, B. R., Mason, J. T., and O'Leary, T. J. (2011). The effect of formaldehyde fixation on RNA: optimization of formaldehyde adduct removal. *The Journal of Molecular Diagnostics : JMD* **13**(3), 282-8, 10.1016/j.jmoldx.2011.01.010.

Karmakar, S., Harcourt, E. M., Hewings, D. S., Scherer, F., Lovejoy, A. F., Kurtz, D. M., Ehrenschwender, T., Barandun, L. J., Roost, C., Alizadeh, A. A., and Kool, E. T. (2015). Organocatalytic removal of formaldehyde adducts from RNA and DNA bases. *Nature Chemistry* **7**(9), 752-8, 10.1038/nchem.2307.

Qiagen (2014). AllPrep DNA/RNA FFPE Handbook.

OP No. NHEERL-H/ISTD/CB/TMM/2012-002-r2. Manual trimming of formalin-fixed paraffin-embedded blocks into tissue sections using the Leica RM2155 Rotary microtome

OP No. NHEERL-ECD-MBC-GK-2007-01. GK NanoDrop nucleic acid quantification.

OP No. NHEERL-H/RTD/GEEBB/DJD/2002-012-002. Analysis of RNA with the Agilent Bioanalyzer and RNA Nano Kit.

**7. APPENDICES**

**Appendix 7.1** Preparation of 40mM Catalyst solution

(To be performed in chemical fume hood)

1. Weigh out 29.94mg of (2-amino-5-methylphenyl)phosphonic acid [fw 187.133] into an 8mL amber glass vial.
2. Add 3.8 mL of nuclease-free water to vial, cap, and place in sonication bath until dissolved.
3. Slowly add 30µL of 0.1 N NaOH solution by drops using micropipette; mix gently.
4. Dip clean glass rod in solution and touch to pH paper – note pH. Rinse rod with water and dry with lab wipe.
5. Repeat steps 3 and 4 as needed to bring solution to pH of 7. Track volume added.
6. Add nuclease-free water to bring solution to final volume of 4mL.
7. Label and store in dark at room temperature.

**Appendix 7.2** Schematic of modified procedure

**Enhanced Qiagen AllPrep Isolation Protocol**

**
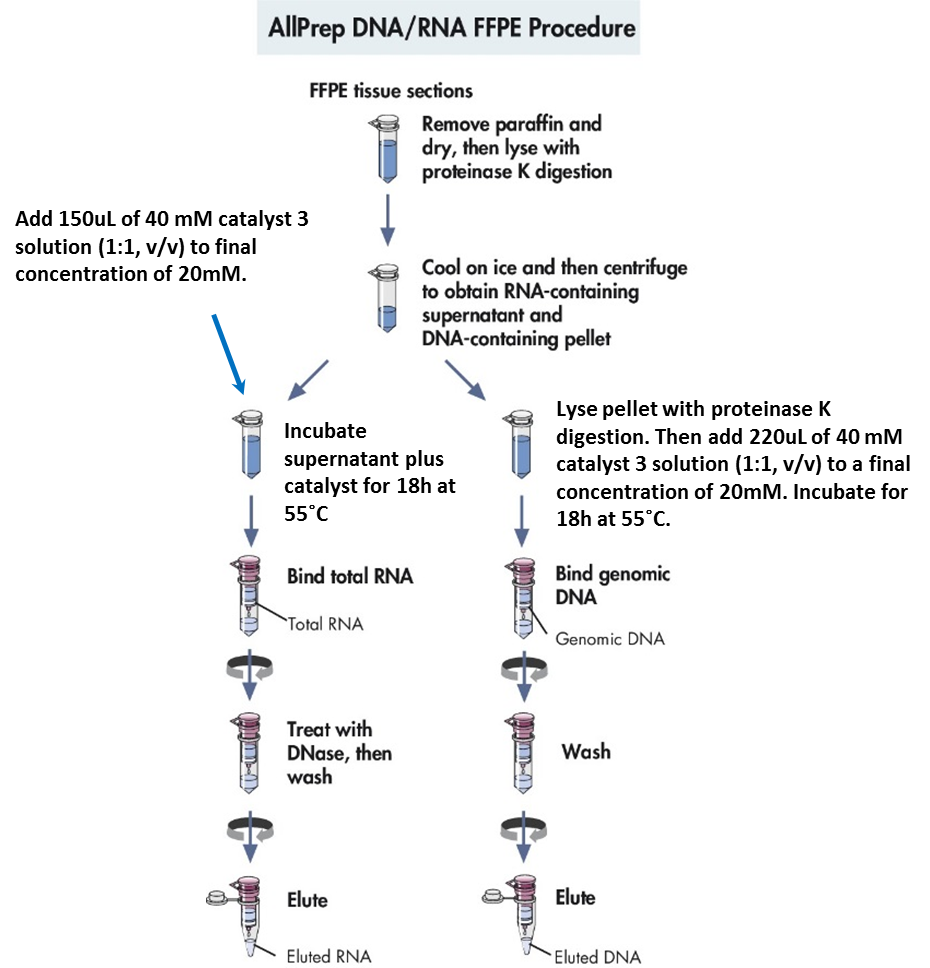
**
